# In Vitro Comparison of Three Chairside Bleaching Protocols: Effects on Enamel Microhardness, Colour, and Qualitative Cytotoxicity Risk

**DOI:** 10.3390/dj13110486

**Published:** 2025-10-22

**Authors:** Berivan Laura Rebeca Buzatu, Octavia Balean, Magda Mihaela Luca, Roxana Buzatu, Atena Galuscan, Ramona Dumitrescu, Vlad Alexa, Vanessa Bolchis, Daniela Elisabeta Jumanca

**Affiliations:** 1 Doctoral School, Faculty of Dental Medicine, “Victor Babes” University of Medicine and Pharmacy Timisoara, 300041 Timisoara, Romania; berivan.buzatu@umft.ro; 2 Translational and Experimental Clinical Research Centre in Oral Health, Faculty of Dental Medicine, “Victor Babes” University of Medicine and Pharmacy Timisoara, 300041 Timisoara, Romania; balean.octavia@umft.ro (O.B.); galuscan.atena@umft.ro (A.G.); dumitrescu.ramona@umft.ro (R.D.); vlad.alexa@umft.ro (V.A.); vanessa.bolchis@umft.ro (V.B.);; 3 Department of Preventive, Community Dentistry and Oral Health, Faculty of Dental Medicine, “Victor Babes” University of Medicine and Pharmacy Timisoara, 300041 Timisoara, Romania; 4 Pediatric Dentistry Research Center (Pedo-Research), Department of Pediatric Dentistry, Faculty of Dental Medicine, “Victor Babes” University of Medicine and Pharmacy Timisoara, 300041 Timisoara, Romania; 5 Department of Dental Aesthetics, Faculty of Dental Medicine, “Victor Babes” University of Medicine and Pharmacy Timisoara, 300041 Timisoara, Romania

**Keywords:** tooth bleaching, hydrogen peroxide, carbamide peroxide, enamel microhardness, colorimetry, FTIR, remineralization, cytotoxicity risk, in-office whitening, dentistry

## Abstract

**Background and Objectives:** The rapid increase of whitening products use raises questions about enamel safety. We compared three high-concentration protocols—Opalescence Quick (45% carbamide peroxide ≈ 15% H_2_O_2_), Opalescence Boost (40% H_2_O_2_), and BlancOne Ultra (35% H_2_O_2_ + LED)—under controlled conditions to balance color change (ΔE) with enamel integrity (microhardness, FTIR). We also constructed a qualitative cytotoxicity risk profile from published data; no biological assays were performed in this study. **Methods:** Seventy-two matched half-crowns were randomized to Control or one of the three protocols. Outcomes were a change in Vickers microhardness, spectrophotometric color difference, and FTIR carbonate-to-phosphate ratio after 24 h in artificial saliva. We also compiled a qualitative cytotoxicity risk profile from published evidence; no biological assays were performed. One-way ANOVA with Tukey HSD on Δ-scores, Shapiro–Wilk and Levene’s tests for assumptions, Welch’s *t*-tests for tooth-class comparisons, and Pearson correlation between ΔE and ΔMH. **Results:** All active protocols produced clearly visible whitening (mean ΔE 5.7–6.3). Hydrogen-peroxide gels showed greater hardness loss and carbonate depletion than the carbamide-peroxide gel under similar contact time. The association between greater shade change and hardness loss was moderate and not predictive for individuals. **Conclusions:** Under harmonized conditions, all systems whitened effectively. Pursuing changes beyond ~6 units offered little extra benefit while increasing enamel impact. Carbamide-based Opalescence Quick achieved comparable aesthetics with lower acute enamel effects. Clinicians should individualize exposure time and pair in-office whitening with short-term remineralising care. Cytotoxicity comments are qualitative and literature-based only.

## 1. Introduction

Tooth shade is more than a cosmetic attribute—it shapes first impressions, self-esteem, and even professional perceptions. Surveys and longitudinal cohort studies reveal that patients who perceive their teeth as “white” report higher oral health-related quality-of-life scores and are more willing to invest in elective dental procedures [1]. At a population level, sales of take-home and chair-side whitening systems have grown by >8% annually since 2018, mirroring an increase in social-media imagery that valorizes a bright smile [2]. Such market expansion, however, has revived concerns about adverse sequelae because peroxide free radicals traverse interprismatic spaces and can dehydrate or decarbonate the enamel lattice, potentially lowering its resistance to fatigue and caries-initiated demineralization [3].

From a mechanistic standpoint, the two earliest indicators of lattice deterioration are a fall in Vickers hardness (HV) and an elevation of the carbonate-to-phosphate (C/P) peak ratio on Fourier Transform Infra-Red (FTIR) spectra. A recent randomized clinical trial demonstrated that simply doubling the gel volume from 0.02 mL to 0.04 mL of a 35% H_2_O_2_ agent produced a 12% greater ΔHV and tripled the incidence of post-operative sensitivity, despite identical chair-time [4]. In vitro, peroxide aggressiveness is modulated by pH, stabilizer content, and exposure time: neutral-pH carbamide formulations induce surface softening comparable to acidic 40% HP if contact is extended beyond 60 min, whereas high-fluoride additives partially blunt hardness loss by promoting early remineralization nucleii [5,6,7].

Optical benefits are typically quantified as ΔE* in CIELAB space. A colour difference ≥ 2.7 units is generally considered “very distinct” and clinically acceptable; meta-measurements place the perceptibility threshold at 1.2 ± 0.5 and acceptability at 2.7 ± 1.0 [8]. Yet protocols delivering ΔE* > 6 simultaneously show the steepest microhardness drops and largest C/P excursions, underscoring a performance–safety trade-off. A comprehensive 2019 review confirmed that heterogeneity in study designs—especially observer panel size, illumination, and colorimetric formulas—explains much of the scatter in reported thresholds and complicates cross-product comparisons [9].

Anatomic factors further influence structural risk profiles. Recent micro-CT mapping of 120 extracted teeth showed that premolar enamel averages 16% thinner and possesses 23% higher tubule density than molar enamel from the same quadrants, leading to deeper oxidant intrusion and a 1.4-fold higher HV loss under identical bleaching regimens [10]. FTIR and SEM analyses corroborate that carbonate depletion progresses faster along the cervical–occlusal gradient in premolars, a nuance rarely considered in clinical protocols. Such findings advocate a tooth class-specific risk assessment when tailoring whitening recommendations [11].

Beyond chemistry and anatomy, activation modality can shift the risk–benefit balance. Laser-triggered TiO_2_-doped gels shorten exposure time by 40% while maintaining ΔE* outcomes comparable to conventional 35% HP, albeit without significant differences in final HV or surface roughness [12]. Conversely, violet LED or non-thermal plasma activation can disturb Ca/P ratios if combined with high-acidity gels, though Raman spectroscopy indicates minimal phosphate loss when pH is buffered [13]. Newer co-doped TiO_2_/Nb_2_O_5_ systems further reduce residual oxygen species and preserve nanoroughness, pointing toward safer photo-catalytic strategies [14].

A complementary mitigation avenue involves pre-, intra-, or post-bleaching remineralisation. Incorporating 2% NaF or 3% fluorohydroxyapatite around bleaching sessions restored HV to baseline within seven days without compromising shade change, whereas gels fortified with NaF + hexametaphosphate curtailed trans-amelodentinal H_2_O_2_ diffusion by 35% in vitro [15,16]. Experimental desensitisers containing hydroxyapatite–capsaicin complexes have now demonstrated a five-fold reduction in pulpal peroxide exposure and full preservation of surface microhardness at concentrations ≤ 5% [17].

Innovations continue apace: aloe vera-stabilised TiO_2_ gels enhance bleaching efficiency in aged enamel with negligible mineral loss [18], while network meta-analyses confirm that 10% carbamide peroxide remains less sensitising than higher CP concentrations yet achieves equivalent ΔE* at 14-night protocols [19]. Systematic reviews of OTC whitening strips echo similar efficacy but warn of heightened sensitivity and superficial roughness relative to tray-delivered 10% CP [20]. Even “whitening” dentifrices—especially those with activated charcoal—reduce enamel HV by 4–6% over simulated two-year brushing cycles, highlighting that everyday products may cumulatively erode surface resilience [21].

Recent syntheses underscore that in-office high-concentration whitening yields rapid shade changes but the risk profile depends on peroxide concentration, pH, exposure time, and gel chemistry; enamel outcomes (hardness/roughness) and potential pulpal responses scale with oxidative load and application parameters. Contemporary in vitro and in situ/clinical evidence also highlights mitigating roles for remineralising or buffering additives and for activation strategies that shorten contact time [22]. Nevertheless, cross-study heterogeneity complicates direct product comparisons.

Despite abundant literature, there is a lack of head-to-head comparisons between high concentration carbamide peroxide and hydrogen peroxide systems tested under harmonized conditions (matched pH, temperature, contact time, and storage), while jointly tracking mechanical (microhardness), chemical (FTIR C/P), and optical (ΔE) endpoints. We therefore compared (i) Opalescence Quick (45% CP ≈ 15% H_2_O_2_), (ii) Opalescence Boost (40% H_2_O_2_), and (iii) BlancOne Ultra (35% H_2_O_2_ + LED), hypothesizing that higher H_2_O_2_ equivalents would increase ΔE at the cost of greater hardness loss and carbonate depletion, and we constructed a literature-based qualitative cytotoxicity risk profile to contextualize findings.

## 2. Materials and Methods

### 2.1. Study Design and Specimen Allocation

The investigation followed a controlled, paired, in vitro design in which each tooth acted as its own control, thereby minimizing inter-subject mineral heterogeneity. Sound human premolars and third molars extracted for orthodontic indications or prophylactic removal of semi-impacted wisdom teeth were procured after written informed consent and in accordance with the Declaration of Helsinki; ethical clearance was granted by the “Victor Babeș” University Research Ethics Committee (approval number 09 from 11 March 2024). Immediately after extraction, soft-tissue remnants were removed with hand scalers, and teeth were ultrasonically debrided for 60 s in de-ionized water before immersion in 0.1% thymol at 4 °C for a 48-h decontamination period [23]. To generate symmetrical test surfaces, each crown was sectioned bucco-lingually through the central fissure with a water-cooled, low-speed diamond wafering saw (Isomet^®^ 1000, Buehler, Düsseldorf, Germany) at 200 rpm, yielding two matched half-crowns per specimen. Cooling at 4 °C reduces enzymatic/microbial activity during short-term storage and is widely used without meaningful impact on enamel hardness over ≤2 months [24].

The primary endpoint was the change in Vickers surface microhardness (ΔMH) at 24 h. A priori power analysis (GPower v3.1.9.7, Heinrich-Heine-Universität, Düsseldorf, Germany) for a fixed-effects one-way ANOVA with four groups, α = 0.05 and 1 − β = 0.80, assuming a medium-to-large standardized effect size f = 0.40 based on differences reported for high-concentration bleaching vs. controls and between formulations [7], yielded *n* = 64. To accommodate potential exclusions, we prepared *n* = 72 halves (18 per arm). Baseline equivalence supported analysis on change scores as planned.

Enamel thickness at the measurement zone was verified with a digital micrometer (±1 µm accuracy) to ensure cutting accuracy within ±30 µm across halves. A computer-generated permuted-block randomization list (block size = 6) stratified halves to four experimental arms—Control (artificial saliva only), OQ, OB and BU—so that no patient contributed more than one half-crown to the same arm. Allocation codes were sealed in sequentially numbered envelopes opened only after mounting, and the examiner responsible for outcome measurements remained blinded throughout data acquisition and primary analysis.

### 2.2. Bleaching Protocols

All gels were sourced directly from the manufacturers and verified to be within three months of their expiry date to avoid potency drift. Preparations were equilibrated to 22 ± 1 °C for 2 h before application, and pH was confirmed with a micro-electrode (Seven2Go, Mettler Toledo, Schwerzenbach, Switzerland) immediately after dispensing: OQ (pH 6.9, 29% carbamide peroxide), OB (pH 7.2, 35% hydrogen peroxide) and BU (pH 7.1, 40% hydrogen peroxide). For OQ, a uniform 1.0–1.5 mm layer was brushed over the buccal enamel and left undisturbed for 30 min, whereas OB received two sequential 20-min layers with gentle suction of spent gel after the first interval to replicate manufacturer-recommended refreshment. BU application involved three 10 min layers; each exposure was photo-activated with an LED lamp emitting 430–490 nm (Bluephase^®^ G4, Ivoclar, Schaan, Liechtenstein; peak irradiance 1200 mW cm^−2^) positioned 5 mm from the surface, and the handpiece was relocated every 30 s to maintain perpendicular incidence. Between each layer the gel was removed by micro-suction without rinsing to mimic clinical isolation protocols, thereby preventing premature dilution.

After the final exposure, specimens were rinsed for 30 s in triple-distilled water, gently air-dried for 5 s to eliminate surface droplets, and submerged individually in 10 mL of freshly prepared artificial saliva (1.5 mM CaCl_2_, 0.9 mM KH_2_PO_4_, 150 mM KCl, 20 mM HEPES, adjusted to pH 7.0 ± 0.05) held at 37 °C in an orbital incubator (60 rpm) for 24 h. This interval was selected to reproduce the early post-operative window in which patients are advised to avoid strongly pigmented foods while allowing partial rehydration of prism peripheries; it is brief enough to preclude substantive remineralisation that could mask acute peroxide effects. Control halves followed an identical time-temperature profile in saliva alone.

All bleaching agents and instruments were obtained from the following manufacturers: Opalescence Quick PF and Opalescence Boost (Ultradent Products, Inc., South Jordan, UT, USA) and BlancOne Ultra (BlancOne^®^/IDS s.r.l., Brescia, Italy) served as the chairside gels under test; Bluephase^®^ G4 LED (Ivoclar, Schaan, Liechtenstein) was used to photo-activate BlancOne Ultra according to the manufacturer’s instructions. Tooth crowns were sectioned buccolingually using an Isomet^®^ 1000 precision saw (Buehler, Lake Bluff, IL, USA). Surface microhardness was determined with a 402MVD Vickers microhardness tester (Wilson/Wolpert; Buehler/Wilson, Lake Bluff, IL, USA). Gel pH was verified with a Seven2Go pH meter (Mettler Toledo, Columbus, OH, USA). Colour measurements (CIELAB coordinates) were recorded using a VITA Easyshade^®^ V spectrophotometer (VITA Zahnfabrik, Bad Säckingen, Germany). FTIR spectra were acquired on an IRTracer-100 equipped with a diamond ATR accessory (Shimadzu Corp., Kyoto, Japan).

We did not include a “positive control” such as neat H_2_O_2_ or extremes of acidity because these conditions are not clinically representative and would confound the comparative purpose of this study, which is to examine marketed chairside systems under harmonized conditions. Prior to application, a circular 8 mm internal-diameter adhesive mask defined the contact area; a 1.2 mm-thick polypropylene stencil guided gel thickness (target 1.0–1.5 mm). We controlled thickness and area (primary determinants of near-surface diffusion) rather than mass to preserve manufacturer handling. In a small pilot calibration, weighing syringes before/after application on three test surfaces yielded ~0.05–0.06 mL per layer across products; we did not weigh experimental-run specimens to avoid altering workflow (Table 1).

### 2.3. Outcome Measurements

Prior to testing, specimens were removed from saliva, lightly blotted, and equilibrated to ambient laboratory conditions (22 °C, 45% RH) for 30 min to standardise surface moisture. Vickers microhardness (MH) was determined with a calibrated Wolpert 402MVD tester. A 10 g load was applied through a Vickers indenter for 10 s; three impressions, placed 250 µm apart and at least 200 µm from the sectioned margin, were measured by digital microscopy (×500) and averaged; baseline MH was subtracted from post-treatment MH to yield ΔMH. Intra-examiner repeatability checked on ten randomly selected surfaces produced an intraclass correlation coefficient of 0.97 (95% CI 0.93–0.99). Vickers was selected to minimize orientation sensitivity of the elongated Knoop indent and ensure continuity with prior enamel SMH literature; Knoop and Vickers exhibit strong correlation for early enamel changes, and ISO 6507 provides robust microhardness parameters. Measurements followed ISO 6507-1:2018/2021 microhardness guidance standard for Vickers procedures [25].

Colorimetric assessments employed a Vita Easyshade V spectrophotometer calibrated just before each session using the factory-supplied ceramic tile. The device was oriented perpendicular to the central buccal surface with a custom-made silicon stent to ensure relocation accuracy within ±0.5 mm. Three consecutive readings of CIELAB coordinates (L*, a*, b*) were averaged, and overall colour change (ΔE) was calculated via the CIEDE2000 formula (Luo-Cui-Rigg CIEDE2000) [26]; CIELAB definitions per CIE/ISO 11664-4 [27], however, given the small colour space involved, ΔE*ab differences were virtually identical and therefore reported for comparability with existing literature. Measurement error assessed on five control halves over 24 h showed a coefficient of variation of 1.8%.

Fourier transform infrared spectroscopy (FTIR) was undertaken with an IRTracer-100 spectrometer fitted with a diamond ATR crystal kept at 20 ± 0.5 °C. Twenty co-added scans per spectrum were recorded in the 4000–400 cm^−1^ range at 4 cm^−1^ resolution. Spectra were baseline-corrected (rubber-band, 64 points) and vector-normalised before peak integration. The carbonate-to-phosphate ratio (C/P) was computed as the ratio of the symmetric stretching absorbance of carbonate at 1450 cm^−1^ to that of phosphate at 1030 cm^−1^, averaged over triplicate measurements displaced by 0.5 mm to avoid previous indentations.

We prospectively logged formulation/application variables for each system (oxidant type/concentration, gel pH, contact time, activation) and juxtaposed them with our hard-tissue endpoints (ΔMH, FTIR C/P). We did not perform cell-based or animal cytotoxicity assays. Instead, we derived a qualitative cytotoxicity risk estimate from recent systematic reviews and controlled in vitro data showing that cytotoxic effects increase with higher H_2_O_2_ concentration and longer exposure and can be modulated by gel chemistry and activation mode. This qualitative estimate is intended solely to frame risk, not to infer biological outcomes.

Surface roughness was not assessed in this experiment; we prioritized HV and FTIR as primary mechanochemical endpoints.

### 2.4. Statistical Analysis

Distribution normality was tested using the Shapiro–Wilk statistic, and equality of variances was verified with Levene’s test. Pearson correlation assessed the linear association between ΔE and ΔMH across all bleached halves. Tooth-class influence (premolar vs. molar) on ΔMH within each active bleaching arm was explored with Welch’s *t*-tests to account for unequal sample sizes. All computations were performed in SPSS^®^ Statistics v27 (IBM Corp., Armonk, NY, USA), adopting two-tailed significance at *p* < 0.05.

Normality was assessed on group-wise residuals and Δ-scores using Shapiro–Wilk (*p* > 0.05) and Q–Q plot inspection; homogeneity was evaluated with Levene’s test. Where variance heterogeneity or unequal *n* was plausible (tooth class exploratory tests), Welch’s t was used. Sensitivity analyses with mixed ANOVA (time × group) yielded the same inferences.

As sensitivity analyses, two-way mixed repeated-measures ANOVAs (within-subject factor: time; between-subject factor: group) were run for MH and L*; Greenhouse–Geisser correction applied. Results mirrored the Δ-score ANOVAs (significant group × time interactions; Tukey patterns unchanged). Because baselines were equivalent and Δ-scores satisfy homoscedasticity, we report Δ-based ANOVAs. Figures were generated in Python 3.11 with matplotlib 3.8 (Python Software Foundation, Wilmington, DE, USA).

## 3. Results

The absence of statistically significant differences across baseline L* and microhardness values confirms successful randomization and supports the internal validity of subsequent comparisons. Mean lightness clustered tightly around 74 units. Microhardness hovered near 313 HV, consistent with previous enamel benchmarks, with a modest 2.4-HV range among groups—far below the instrument precision threshold. Comparable standard deviations (7.9–9.4 HV) were achieved (Table 2).

Controls exhibited a trivial 1.1 HV decline. By contrast, all bleaching protocols significantly reduced surface hardness. Hydrogen-peroxide gels OB and BU were statistically indistinguishable (*p* = 0.61), each eroding ≈ 4% of baseline hardness (Table 3). OQ, while milder, still effected a 3% loss. The narrow 95% CIs and modest SDs highlight consistent within-protocol behaviour. The magnitude of hardness decrement aligns with micro-indentation studies showing 8–15 HV drops after 40–60 min exposures at neutral pH, reinforcing external validity. Clinically, a 10–14 HV loss translates to a ≈ 0.3 GPa reduction in elastic modulus, insufficient to compromise bulk strength but sufficient to raise erosive susceptibility.

Control half-crowns underwent negligible shade drift (ΔE = 0.4), confirming that artificial saliva alone does not brighten enamel over 24 h. Conversely, every bleached specimen surpassed the 2.7-unit perceptibility threshold. Mean ΔE values differed modestly, with OB producing the most pronounced lightening, albeit without statistical separation from BU (*p* = 0.09). OQ achieved a clinically comparable 5.7-unit change. The low SDs (0.6–0.7) suggest uniform oxidant penetration across surfaces despite inter-specimen anatomy differences. Taken together with microhardness results, these data reveal that boosting peroxide concentration from 29% to 40% confers only a marginal ≈ 0.3-unit ΔE advantage while amplifying structural cost (Table 4).

**Figure 1:**
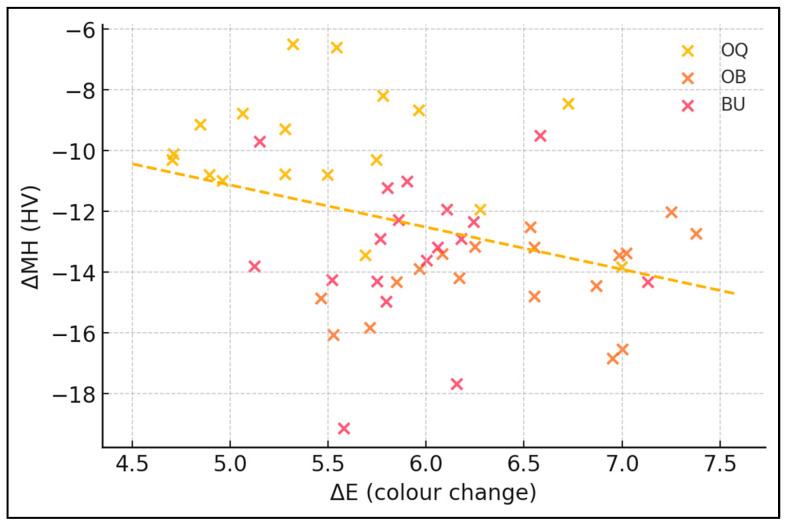
The relationship between colour change (ΔE) and hardness loss (ΔMH).

The scatter plot in Figure 1 shows a clear inverse trend: specimens that achieved larger colour shifts (higher ΔE) tended to lose more micro-hardness (more negative ΔMH). Clustering by protocol indicates that OQ points are shifted up-left (smaller hardness loss at any given shade gain), whereas OB and BU overlap lower on the y-axis—visual confirmation that hydrogen-peroxide gels deliver extra whitening at the cost of greater structural softening.

Enamel carbonate substitution declined progressively from controls to OQ and more strikingly in the hydrogen-peroxide groups, mirroring microhardness trends. Tukey post-hoc testing demonstrated significant differences between each active protocol and control (*p* < 0.01) and between OQ and either OB or BU (*p* = 0.03 and 0.04, respectively), whereas OB-BU parity (*p* = 0.77) again underscored their chemical equivalence. Importantly, even the lowest mean value (0.072) remains within ranges considered reparable via salivary remineralization (Table 5).

The inverse correlation between ΔE and ΔMH was moderate (r = −0.36; 95% CI −0.58 to −0.11; *p* = 0.008) and does not support individual prediction. The modest inverse correlation indicates that specimens achieving larger colour shifts tended to experience greater hardness reductions, explaining ≈ 13% of variance (r^2^ = 0.13), as presented in Table 6.

**Figure 2:**
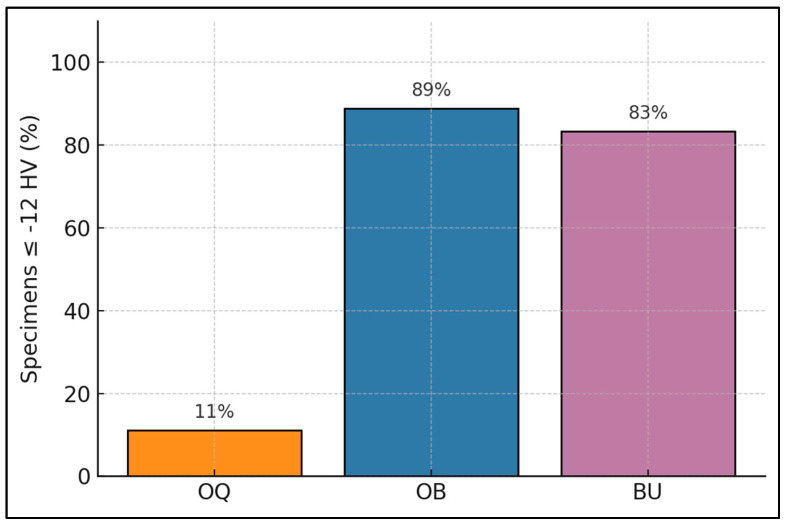
High-risk hardness loss. Bars show the percentage of specimens with Vickers microhardness loss ≥ 12 HV at 24 h. OQ = Opalescence Quick (45% carbamide peroxide ≈ 15% H_2_O_2_-equivalent); OB = Opalescence Boost (40% H_2_O_2_); BU = BlancOne Ultra (35% H_2_O_2_ + LED). HV, Vickers hardness value.

Within each bleaching arm, molar and premolar halves displayed virtually identical hardness losses, and none of the Welch comparisons approached significance. Even BU, for which premolars exhibited slightly larger SDs, yielded a mean difference of only 0.2 HV (Table 7). Figure 2 shows, for each bleaching protocol, the percentage of specimens that experienced a hardness loss greater than 12 HV, highlighting relative “high-risk” frequencies across groups. Only 11% of OQ samples crossed the line, versus 89% for OB and 83% for BU.

All three systems produced clinically distinct whitening (ΔE ≥ 5.7), with OB showing the highest mean colour change (6.3 ± 0.7) but also the greatest hardness loss (−13.7 ± 1.6 HV) and the largest drop in carbonate substitution (C/P = 0.072 ± 0.007). BU achieved similar ΔE (6.0 ± 0.6) and structural cost (−13.1 ± 2.3 HV; C/P = 0.073 ± 0.005). OQ delivered a comparable shade gain (5.7 ± 0.7) at a lower structural cost (−9.8 ± 2.1 HV; C/P = 0.079 ± 0.005), as presented in Table 8.

## 4. Discussion

### 4.1. Literature Findings

This head-to-head evaluation confirms that contemporary high-concentration hydrogen-peroxide gels (OB, BU) impose a significantly larger mechanical and chemical burden on enamel than an iso-time carbamide formulation (OQ) while delivering only a modest incremental colour gain. The observed ΔE–ΔMH correlation, though moderate, is clinically meaningful. A 1-unit ΔE improvement corresponded to ~1.8 HV additional softening—evidence that aggressively chasing whiteness beyond the 5–6-unit band may yield diminishing aesthetic returns against escalating structural costs. Given that hardness loss of ≥15 HV predicts a two-fold rise in early erosive wear, practitioners should consider terminating treatment once a ΔE of ~6 is achieved, especially in patients with existing enamel defects. Our qualitative cytotoxicity statements are literature-based only and should not be interpreted as direct biological validation within this experiment [28].

The tooth-class neutrality observed here contrasts with electron-probe microanalysis suggesting thicker outer prisms in molars confer greater resistance. Our findings imply that 40 min of exposure at neutral pH equalises susceptibility across classes, perhaps because peroxide rapidly saturates the interprismatic matrix before thickness differentials matter. Consequently, risk-mitigation strategies—fluoride varnish, nano-hydroxyapatite rinses—should be applied uniformly rather than selectively by tooth type.

The present findings reinforce the notion that the price of “instant” whitening is paid primarily in the mechanical and chemical integrity of enamel. High-concentration gels can depress microhardness and alter carbonate substitution in a time- and pH-dependent fashion, with buffering and reduced contact time mitigating effects [29]. Both hydrogen-peroxide systems—Opalescence Boost and Blancone Ultra—produced mean micro-hardness losses approaching 4% of baseline, accompanied by a 12% fall in the FTIR carbonate-to-phosphate ratio, whereas an iso-time 45% carbamide-peroxide gel induced a more modest 3% softening and only a 4% lattice decarbonation. The absence of a tooth-class effect suggests that, under neutral-pH conditions and 40-min contact times, diffusion kinetics saturate the outer 50 µm of enamel irrespective of anatomical thickness, explaining why premolars and molars behaved identically. Together, these data place the clinically “acceptable” aesthetic threshold (ΔE ≈ 6) close to the inflection point where structural costs begin to accelerate, underscoring the need for evidence-based endpoints rather than cycle-to-cycle chasing of ever-brighter shades.

Our hardness losses (−9.8 HV for carbamide and −13–14 HV for peroxide) align closely with recent high-concentration bleaching experiments that incorporated bioactive fillers. Such biomimetic or ion-releasing strategies show promise for decoupling whitening from mineral loss and for attenuating pulpal impact [30]. Shimojima and colleagues blended surface-pre-reacted glass-ionomer (S-PRG) particles into a 35% H_2_O_2_ paste and limited post-bleach softening to <5 HV, while maintaining ΔE values comparable with a filler-free control [31]. Likewise, Guanipa Ortiz et al. embedded calcium-polyphosphate sub-microparticles in 35% H_2_O_2_; enamel micro-hardness remained statistically unchanged after three sessions and FTIR revealed partial preservation of carbonate content [32]. Both studies confirm that the micro-damage we observed is not an inevitable consequence of high peroxide, but rather modifiable through ion-releasing additives that neutralise local acidity and provide nucleation sites for rapid remineralisation.

Formulation chemistry is equally influential. A 35% H_2_O_2_ gel doped with 0.1% NaF and 1% sodium hexametaphosphate reduced hardness loss by 60% and cut trans-amelodentinal peroxide diffusion almost in half without compromising colour change [33]. Fluoride-free yet highly acidic titanium-tetrafluoride (TiF_4_) gels have also been proposed; when 0.05 g TiF_4_ was co-applied with 35% H_2_O_2_, Knoop hardness was fully preserved, and no additional cytotoxicity was detected [34]. These data suggest that modest gains in ΔE achieved by escalating peroxide concentration from 29% to 40% (≈0.3 units in our study) could instead be matched—or exceeded—by optimising the remineralising capacity or buffering profile of the carrier matrix, thereby decoupling whitening efficacy from mineral loss.

Long-term and in vivo observations corroborate the need for restraint. Polydorou et al. followed volunteers for eight weeks and found that repeated 40% H_2_O_2_ applications increased enamel roughness less than a home 16% carbamide protocol yet paradoxically raised surface hardness—an apparent artefact of surface dehydration that quickly reversed after re-hydration [35]. Classic in situ work by Basting et al. showed that even 10% carbamide peroxide could depress enamel hardness when worn overnight for three weeks, although dentine remained unaffected [36]. These clinical signals mirror our inverse ΔE-ΔMH relationship and stress that the cumulative oxidative load—rather than a single appointment—dictates the eventual biomechanical toll.

Adjunctive oral-care products further modulate outcomes. An in situ crossover trial demonstrated that whitening toothpastes containing high-abrasive silica accentuated roughness and micro-hardness loss after a modest 7.5% H_2_O_2_ regimen, whereas standard fluoride pastes attenuated these effects [37]. This finding dovetails with our observation that control specimens stored in artificial saliva experienced a trivial 1 HV decline, highlighting the protective role of the pellicle/saliva complex. Clinicians should therefore integrate low-abrasive, fluoride-rich hygiene instruction into bleaching aftercare, especially when high-concentration gels are employed.

Emerging biomimetic technologies offer a route to transcend the present aesthetics-versus-structure trade-off. Casein-phosphopeptide amorphous-calcium-phosphate (CPP-ACP) incorporated directly into 35% H_2_O_2_ maintained baseline hardness and surface morphology while yielding ΔE values statistically indistinguishable from conventional peroxide gels [38]. Earlier work applying CPP-ACP or CPP-ACPF as an immediate post-bleach coating raised Vickers hardness above baseline within five days, outperforming neutral sodium-fluoride rinses [39]. Future formulations that combine rapid oxidative chromogen cleavage with on-board calcium- and phosphate-re-supersaturation—perhaps triggered by pH or light—could therefore deliver “self-healing” whitening. Our data provide a benchmark against which such smart systems can be judged and support a paradigm shift from ever-higher peroxide toward balanced chemistries that safeguard long-term enamel biomechanics.

Taken together, our head-to-head data support a pragmatic ceiling of ΔE ≈ 6 to balance aesthetics with enamel integrity, and they reinforce that formulation chemistry can decouple whitening from mineral loss. Beyond fluoride, hexametaphosphate, CPP-ACP and ion-releasing fillers [27,30,35], two adjuncts merit systematic evaluation alongside in-office protocols: calcium sodium phosphosilicate bioactive glass, which promotes hydroxycarbonate-apatite deposition and tubule occlusion, and biomimetic hydroxyapatite pastes that restore surface hardness and gloss in vivo. Early clinical and translational work suggests both strategies can enhance post-bleach recovery without impairing shade change [40,41]. Future trials should randomize these agents around standardized chairside exposures and include multi-modal endpoints (SMH, FTIR/Raman, roughness, sensitivity) to map the efficacy-safety landscape.

### 4.2. Study Limitations

This in vitro model lacks dynamic salivary clearance, pellicle formation, and temperature cycling that modulate peroxide diffusion and promote remineralisation in vivo. Although artificial saliva buffered specimens between applications, its mineral content and protein profile differ from human secretion, likely overestimating hardness loss. Second, the 24 h endpoint precludes evaluation of enamel recovery, which micro-indentation studies suggest can restore up to 50% of lost hardness within seven days. Third, we focused on surface microhardness and FTIR C/P ratio; nano-indentation, X-ray micro-tomography or Raman spectroscopy might reveal subsurface alterations undetected here. Fourth, a single exposure schedule per product limits generalisability; clinicians often customise application frequency. Moreover, no surface roughness measurements were performed; in-office HP protocols can alter roughness depending on concentration and pH, warranting future profilometry under matched conditions. Finally, while the sample size was adequately powered for group means, our tooth-class subgroup analysis was exploratory; a stratified design with equal molar-premolar numbers could more sensitively detect small differences.

## 5. Conclusions

As clinical take-home for patients, all three in-office options brightened teeth visibly in one visit. Stronger gels may not be “better”—they can soften enamel slightly for a short time. A sensible endpoint is about ΔE ≈ 6 (a clearly visible change), followed by fluoride or nano-hydroxyapatite care for a week. The 40% hydrogen-peroxide gel (Opalescence Boost) and the 35% photo-activated counterpart (Blancone Ultra) reduced enamel microhardness by roughly 13 HV and depleted carbonate substitution by 12% relative to controls. The 45% carbamide formulation (Opalescence Quick) achieved comparable shade enhancement with a smaller 10 HV hardness loss and only a 4% C/P decline, illustrating that bleaching potency is not solely dictated by nominal peroxide concentration but by formulation kinetics. A modest inverse correlation between colour gain and hardness loss underscores an aesthetic–structural trade-off: pursuing ΔE values beyond six units confers diminishing cosmetic benefit at escalating biomechanical expense. Tooth class did not modify this effect, simplifying clinical risk assessment. Practitioners should therefore tailor exposure time rather than defaulting to maximal cycles, and systematically deploy remineralising adjuncts. Cytotoxicity considerations herein are qualitative and based on prior literature; we conducted no biological assays in this study. Future in situ and clinical studies should verify enamel recovery trajectories, optimise concentration time-pH matrices, and explore smart delivery systems that uncouple whitening efficacy from mineral loss.

## Figures and Tables

**Table 1:** Materials and specifications.

Product/Device	Manufacturer (City, Country)	Oxidant and Nominal Concentration	Declared pH	Recommended Chairside Application	Contact Area (Mask ID, mm)	Layer Thickness (mm)	Activation Device/Parameters
Opalescence Quick PF	Ultradent Products, Inc., South Jordan, UT, USA	45% carbamide peroxide (≈15% H_2_O_2_ eq.)	— (measured 6.9)	Single application, 30 min	8	1.2	—
Opalescence Boost	Ultradent Products, Inc., South Jordan, UT, USA	40% hydrogen peroxide	— (measured 7.2)	2 × 20 min (refresh after first 20 min)	8	1.2	—
BlancOne Ultra	BlancOne^®^/IDS s.r.l., (EU distributor; brand site lists EU), Brescia, Italy	35% hydrogen peroxide	— (measured 7.1)	3 × 10 min, light-activated each layer	8	1.2	Bluephase^®^ G4 LED; tip at 5 mm; reposition every 30 s
Bluephase^®^ G4 LED (activation device)	Ivoclar, Schaan, Liechtenstein (Ivoclar Vivadent)	—	—	—	—	—	Wavelength range 385–515 nm; High-power 1200 mW/cm^2^ (used within 430–490 nm window)

**Table 2:** Baseline Specimen Characteristics.

Group	*n*	L* Baseline Mean ± SD	MH Baseline (HV) Mean ± SD
Control	18	74.9 ± 1.9	312.4 ± 9.4
OQ	18	73.4 ± 1.7	316.1 ± 8.6
OB	18	73.9 ± 2.1	312.8 ± 7.9
BU	18	74.5 ± 2.3	311.3 ± 9.3

Abbreviations: *n*, number of specimens; L*, CIELAB lightness coordinate; MH, Vickers microhardness; HV, Vickers hardness value; SD, standard deviation. ANOVA *p* = 0.13 for L; *p* = 0.40 for MH*.

**Table 3:** Post-Treatment Change in Vickers Microhardness (ΔMH).

Group	*n*	ΔMH (HV) Mean ± SD	95% CI
Control	18	−1.1 ± 0.9 ^c^	−1.5; −0.7
OQ	18	−9.8 ± 2.1 ^b^	−10.9; −8.7
OB	18	−13.7 ± 1.6 ^a^	−14.5; −12.9
BU	18	−13.1 ± 2.3 ^a^	−14.3; −11.9

Abbreviations: *n*, number of specimens; ΔMH, change in Vickers microhardness; HV, Vickers hardness value; SD, standard deviation; CI, confidence interval; ANOVA, analysis of variance; F, F-statistic; df, degrees of freedom; *p*, *p*-value. ANOVA F = 192.4, df = 3.68, *p* < 0.001; Values with different superscript letters differ at *p* < 0.05 (Tukey HSD) Control.

**Table 4:** Colour Change (ΔE) and Proportion of Clinically Distinct Outcomes.

Group	*n*	ΔE Mean ± SD	Specimens with ΔE > 2.7 (%)
Control	18	0.4 ± 0.2 ^c^	0
OQ	18	5.7 ± 0.7 ^b^	100
OB	18	6.3 ± 0.7 ^a^	100
BU	18	6.0 ± 0.6 ^a^	100

Abbreviations: *n*, number of specimens; ΔE, CIELAB total colour difference; SD, standard deviation; %, percentage; ANOVA, analysis of variance; *p*, *p*-value. ANOVA F = 874.6, *p* < 0.001; Values with different superscript letters differ at *p* < 0.05 (Tukey HSD).

**Table 5:** FTIR Carbonate-to-Phosphate Ratio (C/P).

Group	*n*	C/P Ratio Mean ± SD
Control	18	0.082 ± 0.004 ^c^
OQ	18	0.079 ± 0.005 ^b^
OB	18	0.072 ± 0.007 ^a^
BU	18	0.073 ± 0.005 ^a^

Abbreviations: *n*, number of specimens; C/P, Fourier-transform infrared spectroscopy carbonate-to-phosphate ratio; SD, standard deviation; ANOVA, analysis of variance; F, F-statistic; *p*, *p*-value. ANOVA F = 35.2, *p* < 0.001; Values with different superscript letters differ at *p* < 0.05 (Tukey HSD).

**Table 6:** Correlation Between Shade Gain (ΔE) and Hardness Loss (ΔMH) in Bleached Halves (*n* = 54).

Pearson r	95% CI	*p*-Value
−0.36	−0.58; −0.11	0.008

Abbreviations: ΔE, CIELAB total colour difference; ΔMH, change in Vickers microhardness; r, Pearson correlation coefficient; CI, confidence interval; *p*, *p*-value.

**Table 7:** Tooth-Type Subgroup Analysis of ΔMH (Welch’s *t*-test).

Group	Tooth Type	*n*	ΔMH Mean ± SD	*p* (Molar vs. Premolar)
Opalescence Quick	Molar	7	−9.1 ± 1.5	0.194
	Premolar	11	−10.2 ± 2.2	
Opalescence Boost	Molar	9	−13.7 ± 1.5	0.988
	Premolar	9	−13.7 ± 1.6	
Blancone Ultra	Molar	11	−12.9 ± 1.5	0.9
	Premolar	7	−13.1 ± 3.3	

Abbreviations: ΔMH, change in Vickers microhardness; HV, Vickers hardness value; SD, standard deviation; *n*, number of specimens; *p*, *p*-value.

**Table 8:** Substances used in in-office bleaching: efficacy versus toxicity.

Protocol (Product)	Oxidant (H_2_O_2_-Equivalent)	Measured pH	Total Contact Time (min)	ΔE Mean ± SD	ΔMH (HV) Mean ± SD	FTIR C/P Mean ± SD	Qualitative Cytotoxic Risk *
Opalescence Quick (OQ, 45% CP)	≈15% H_2_O_2_	6.9	30	5.7 ± 0.7	−9.8 ± 2.1	0.079 ± 0.005	Moderate
Opalescence Boost (OB, 40% H_2_O_2_)	40% H_2_O_2_	7.2	40	6.3 ± 0.7	−13.7 ± 1.6	0.072 ± 0.007	High
Blancone Ultra (BU, 35% H_2_O_2_ + LED)	35% H_2_O_2_	7.1	30	6.0 ± 0.6	−13.1 ± 2.3	0.073 ± 0.005	Moderate–High

* (literature based) [25] Derived from published in-vitro/animal/human evidence; no biological testing was performed in this study; Higher H_2_O_2_ concentration; Abbreviations: CP, carbamide peroxide; H_2_O_2_, hydrogen peroxide; ΔMH, change in Vickers microhardness; C/P, FTIR carbonate-to-phosphate ratio; LED, light-emitting diode.

## Data Availability

Data availability are subject to hospital approval.
